# The microbial biodiversity at the archeological site of Tel Megiddo (Israel)

**DOI:** 10.3389/fmicb.2023.1253371

**Published:** 2023-09-22

**Authors:** Yali Zhang, S. Emil Ruff, Nikolay Oskolkov, Braden T. Tierney, Krista Ryon, David Danko, Christopher E. Mason, Eran Elhaik

**Affiliations:** ^1^Department of Biology, Lund University, Lund, Sweden; ^2^The Marine Biological Laboratory, Woods Hole, MA, United States; ^3^Department of Biology, National Bioinformatics Infrastructure Sweden, Science for Life Laboratory, Lund University, Lund, Sweden; ^4^Department of Physiology and Biophysics, Weill Cornell Medicine, New York, NY, United States; ^5^The HRH Prince Alwaleed Bin Talal Bin Abdulaziz Alsaud Institute for Computational Biomedicine, New York, NY, United States; ^6^The Feil Family Brain and Mind Research Institute (BMRI), New York, NY, United States; ^7^The Information Society Project, Yale Law School, New Haven, CT, United States; ^8^The WorldQuant Initiative for Quantitative Prediction, Weill Cornell Medicine, New York, NY, United States

**Keywords:** microbiome, pathogens, biocorrosion, monuments, acid-producing bacteria (APB), Urban microbiome, Monumentome, MetaSUB

## Abstract

**Introduction:**

The ancient city of Tel Megiddo in the Jezreel Valley (Israel), which lasted from the Neolithic to the Iron Age, has been continuously excavated since 1903 and is now recognized as a World Heritage Site. The site features multiple ruins in various areas, including temples and stables, alongside modern constructions, and public access is allowed in designated areas. The site has been studied extensively since the last century; however, its microbiome has never been studied. We carried out the first survey of the microbiomes in Tel Megiddo. Our objectives were to study (i) the unique microbial community structure of the site, (ii) the variation in the microbial communities across areas, (iii) the similarity of the microbiomes to urban and archeological microbes, (iv) the presence and abundance of potential bio-corroding microbes, and (v) the presence and abundance of potentially pathogenic microbes.

**Methods:**

We collected 40 swab samples from ten major areas and identified microbial taxa using next-generation sequencing of microbial genomes. These genomes were annotated and classified taxonomically and pathogenetically.

**Results:**

We found that eight phyla, six of which exist in all ten areas, dominated the site (>99%). The relative sequence abundance of taxa varied between the ruins and the sampled materials and was assessed using all metagenomic reads mapping to a respective taxon. The site hosted unique taxa characteristic of the built environment and exhibited high similarity to the microbiome of other monuments. We identified acid-producing bacteria that may pose a risk to the site through biocorrosion and staining and thus pose a danger to the site’s preservation. Differences in the microbiomes of the publicly accessible or inaccessible areas were insignificant; however, pathogens were more abundant in the former.

**Discussion:**

We found that Tel Megiddo combines microbiomes of arid regions and monuments with human pathogens. The findings shed light on the microbial community structures and have relevance for bio-conservation efforts and visitor health.

## Background

Tel (mound) Megiddo is regarded as one of Israel’s most important archeological sites for the Bronze and Iron Ages (Finkelstein et al., 2000). The site is situated at the fertile Jezreel Valley in northern Israel. It is strategically located close to a water supply on a hill (ca. 20 m) above sea level and along a major road that connected ancient Egypt to Mesopotamia. It has been suggested that Megiddo served as an important military base and an emporium established by the Northern Kingdom for training and trading horses for military purposes (Cantrell and Finkelstein, 2006). The international importance of Megiddo is attested in several ancient texts from the Near East to North Africa. Megiddo is mentioned several times in the Bible and is the only site in the Levant mentioned in the written texts of all the neighboring civilizations, including the Egyptians (Faulkner, 1942), Hittites (Hattians) (Singer, 1995), and Assyrians (Fales and Postgate, 1995).

The mound has undergone multiple excavations, beginning in the last century with Gottlieb Schumacher, Yigael Yadin, and the Oriental Institute of the University of Chicago, which uncovered rich findings attesting to the value of the site (Finkelstein et al., 2000). The mound harbors the remains of 30 settlements dating from the Neolithic (7th millennium BC) to the Persian period (4th century BC) (Ussishkin, 2015). It includes sediment resulting from fill or debris deposits, remains of vegetal deposits, soilbased construction materials and their degradation products (including mud bricks, degraded mudbrick material, floor surfacing, and wattleanddaub roof construction), limestone or chalkbased construction materials, or burned vegetal matter (Regev et al., 2015). The walls in the mound are similarly constructed, at least partially, from limestones, lime plastered with various flimsy installations, and mud bricks covered with chalk wall plaster (Regev et al., 2015; Ussishkin, 2015). These excavations utilized multiple techniques to study various aspects of the inhabitants and their environment. They positioned Tel Megiddo as a fundamental site for understanding the culture and chronology of the Canaanites, Israelites, and Assyrians (e.g., Finkelstein et al., 2022). Thus far, however, the microbiomes of the site have not been studied, leaving open many questions about the microbial communities on the site, their classification, structure, biodiversity, and potential health risks to visitors. It is also well established that acid-producing bacteria (APB) represent a risk to archeological sites because the microbes produce acidic metabolites that decrease the pH of their environment, corroding and damaging stone monuments and artifacts. However, due to the lack of data, it is unknown whether APBs exist on the site.

Here, we report the first cartographic effort of the urban and soil microbiome at Tel Megiddo.

We collected 40 swab samples from 10 major areas in the site and identified microbial taxa using next-generation sequencing to study four major topics: (i) The microbial diversity at the different areas in the site; (ii) the similarity of the Tel Megiddo microbiomes to the microbiomes of the urban environment or other archeological sites. Much like urban transit systems, Tel Megiddo is a center of societal interactions, hosting hundreds of thousands of visitors every year. International travelers bring their commensal microorganisms to the public open areas on the site, and they come into contact with microorganisms on the site. It is unclear what effect visitors have on the microbial communities of the site; (iii) the presence and abundance of APBs; and (iv) the presence and abundance of potentially pathogenic microbes. The urban environment harbors with thousands of microorganisms, some of which are pathogens that exchange antimicrobial resistance (AMR) markers linked to pathogenicity (Danko et al., 2021). Our findings uncover the hidden world of microorganisms at Tel Megiddo and highlight potential risks to the site and visitors.

## Results

This is the first large-scale study of the structure and diversity of the microbial communities in the archeological site of Tel Megiddo (Israel), which was carried out in July 2018. During the winter season, the region experiences average temperatures ranging from 6°C to 26°C, along with a high relative humidity (39–59%) and an average precipitation of 450 mm. The summer season is characterized by average temperatures ranging from 14–34°C with a relative humidity of 37–41% and no precipitation. During July, daytime temperatures averaged 31 to 32°C, while nighttime temperatures ranged from 21 to 23°C at night, and the relative humidity was 41%.^1^^,^ ^2^

**Figure 1:**
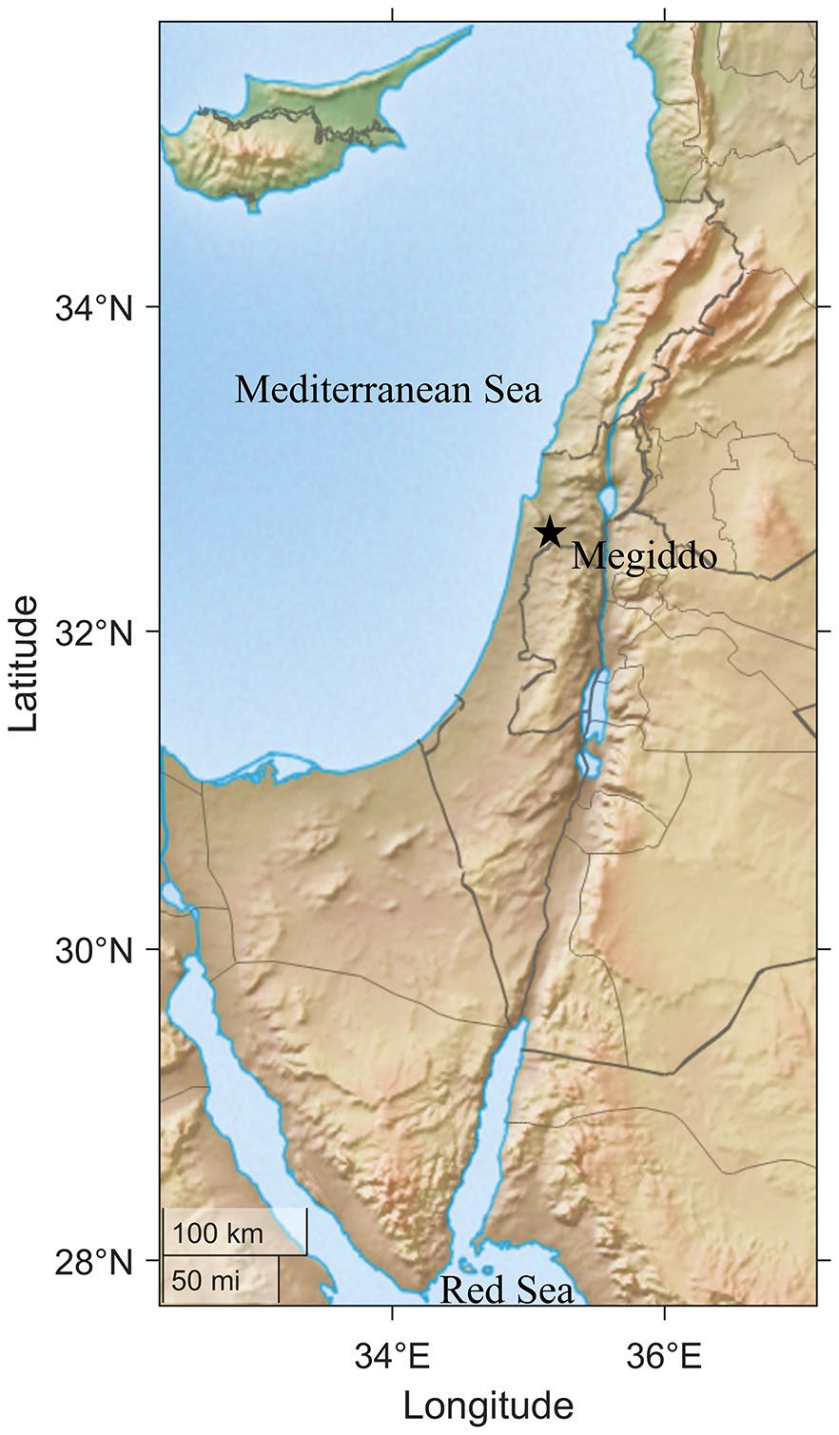
Map of the southern Levant and the site of Megiddo.

We collected 40 swab samples from surfaces in 10 areas within Tel Megiddo, including one location 50 meters outside the archeological site as an outgroup (Supplementary Table S1). Our sampling covered the visitor entrance and all major archeological areas of the site (Figure 1), including CC (the Southern Palace), J (Temples), and the two-chambered city gate, and included various materials, like basalt and stone. Each sample was sequenced with 5–7 million 125 bp paired-end reads using Illumina NGS sequencers.

We identified 4,259 microbial lineages (Supplementary Table S2). We determined that none of the sampled organisms were ancient by evaluating the deamination pattern and the read length distribution quality metrics (see Methods). Most of the taxa (83%) could be taxonomically annotated (Supplementary Table S3). The overall average relative sequence abundance (RSA) of bacteria was 77.6%, with a minority of viruses (0.0006%) and archaea (0.2%). The remaining sequences could not be assigned (Supplementary Figure S1). The most abundant phyla were *Actinobacteria* (~53%) and *Proteobacteria* (~35%) (Supplementary Table S4A). Of all bacteria, 242 (5.7%) were annotated as pathogens (Supplementary Table S5A), including 172 animal and 59 plant pathogens, with 11 additional pathogens infecting both hosts.

### Microbial communities include unique and common taxa

**Figure 2:**
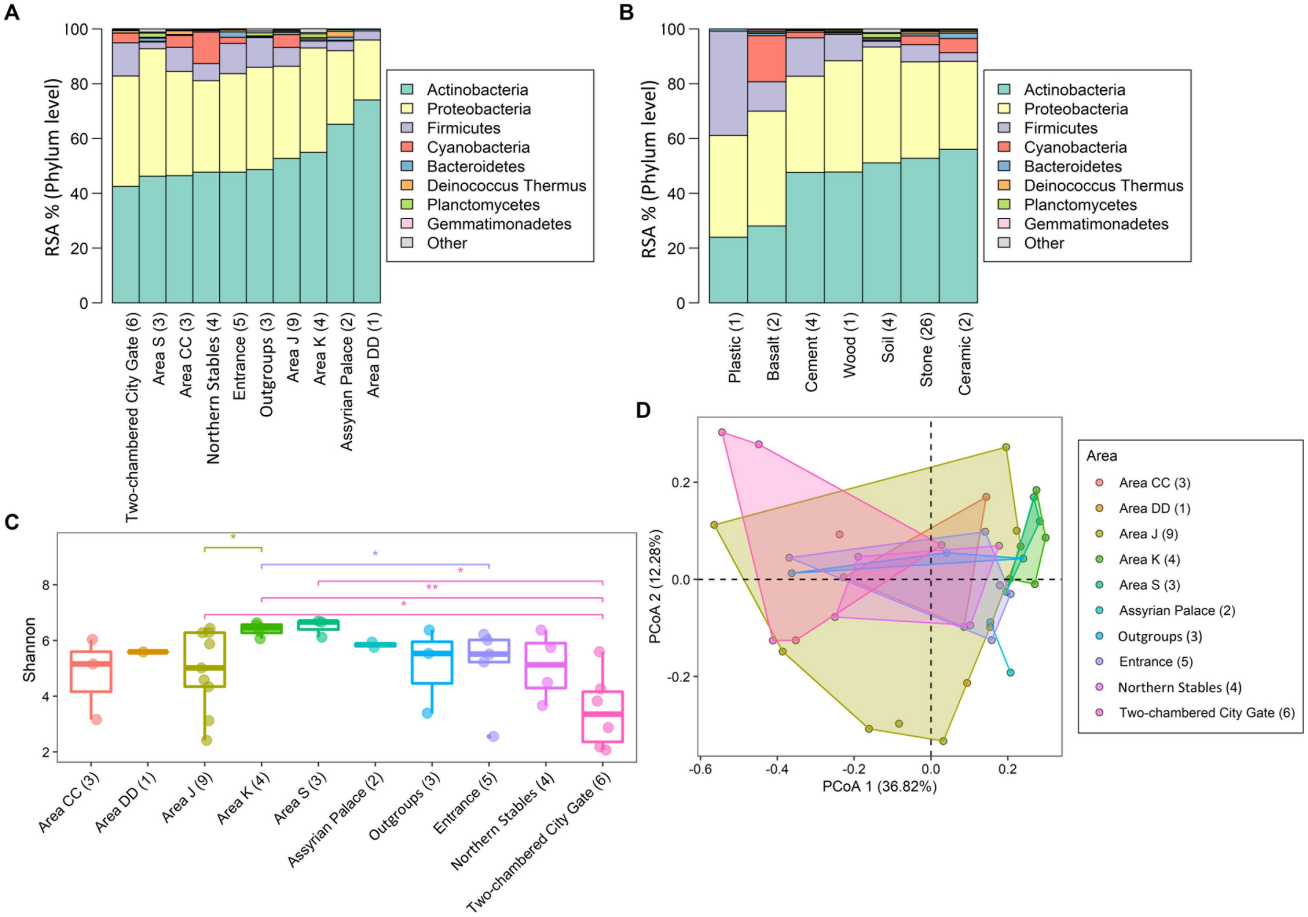
Distribution of taxa prevalence in different areas of Tel Megiddo. The number and percentage of taxa classified into phyla in different areas are shown. 428 bacterial taxa occur in all areas (100%), representing the core microbiome. 583 bacterial taxa occur in only one area, thus being unique to a specific part of Tel Megiddo.

Concerning the prevalence of taxa at the site, of the 3,418 bacteria, 13% were present across all areas (nine Tel Megiddo areas and one outgroup site), while 17% were exclusive to each specific area (Figure 2 and Supplementary Table S2). Area J had the largest number of unique bacteria (470), followed by the northern stables (45) and Area CC (41). By contrast, Area DD, the Assyrian Palace, and the two-chambered city gate had no unique bacteria. Of the microbiomes unique to each Tel Megiddo area, the five phyla with the lowest RSA include *Caldiserica* (Area S), *Candidatus Saccharibacteria* (northern stables), and *Elusimicrobia* (Area J). Interestingly, the three areas with the lowest normalized RSA’s standard deviation of the most common phyla (18.8–18.2%) were the most publicly accessible ones (Supplementary Table S4A) and included Area CC, visitor entrance, northern stables, and the two-chambered city gate, followed by the outgroup samples, whereas the least accessible area: Area DD exhibited the highest standard deviation (26%). These results indicate that if left undisturbed, the microbiomes of each area may become more dissimilar potentially due to environmental factors like the specific material of the site, exposure to sun and rain, and interactions with other taxa.

The most abundant taxa unique to To Tel Megiddo, i.e. not present in other urban or monumentome datasets, were (Supplementary Table S7): *Mycoplasma haemofelis*, an animal pathogen that can be disseminated by blood-sucking arthropod vectors, which may be transmissible to humans (Pires dos Santos et al., 2008), *Candidatus Kinetoplastibacterium galatii* the endosymbionts of strigomonas and angomonas, *Acetohalobium arabaticum*, which, to the best of our knowledge, has only been isolated from the lagoons of the Arabat spit (East Crimea) (Prat et al., 2012), and *Avibacterium paragallinarum*, a pathogen of chickens and birds (Guo et al., 2022).

**Figure 3:**
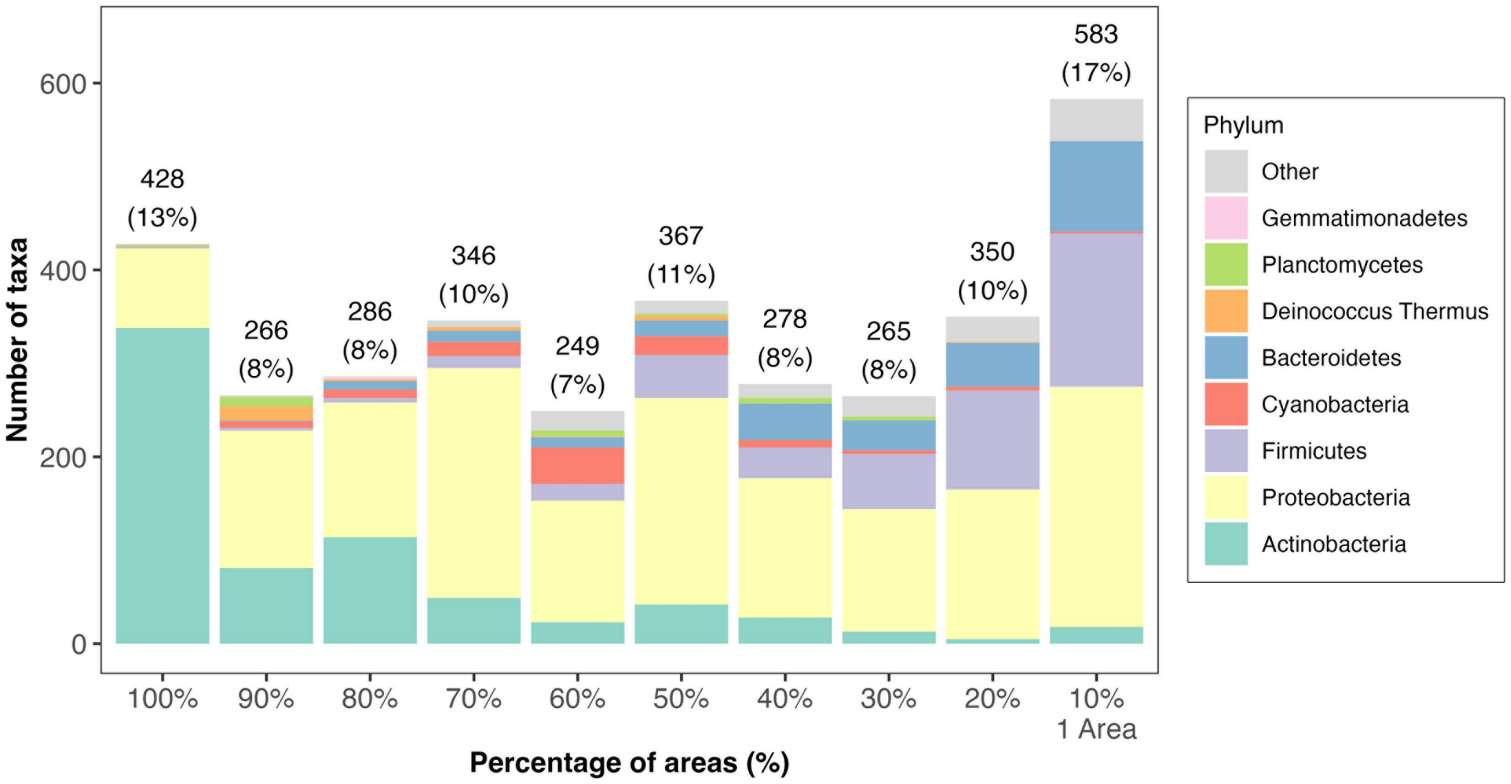
Microbial diversity in different areas and materials at the phylum level. The taxonomic composition of the taxa varied between samples found in different areas **(A)** and surface materials **(B)**. “Other” refers to all other phyla with lower RSA than the displayed phylum. Subfigures show the RSA (%) **(A,B)** of the top eight most abundant phyla and the Shannon index of the microbial communities **(C)**. Significance was assessed using the Wilcoxon signed-rank test with the *p*-value marked in the plots as 0–0.001***, 0.001–0.01**, 0.01–0.05* or NS (non-significant difference), and principal component analyses based on Bray-Curtis distances of the microbial communities in different sites **(D)** *PERMANOVA test* (*r* = 0.38 ± 0.03, *p* = 0.04 ± 0.04) Convex hull function was used to draw the clusters.

Six bacterial phyla, *Actinobacteria*, *Proteobacteria*, *Bacteroidetes*, *Firmicutes*, *Gemmatimonadetes*, and *Planctomycetes*, were found across all the areas (Supplementary Table S4A). Together with *Cyanobacteria* and *Deinococcus Thermus* (though both were absent from Area DD), they comprised over 99% of all annotated phyla in Tel Megiddo (Figure 3A). Normalizing the RSA for the eight phyla (Supplementary Table S4A), the predominant phylum was *Actinobacteria* (43–74% RSA), mainly represented by the orders *Actinomycetales* (4–27%) and *Propionibacteriales* (0.5–18%) and the taxa *Tessaracoccus* sp. *T2.5–30* (0–12%), *Kytococcus sedentarius* (0–7%), and *Modestobacter marinus* (0–13%). The orders *Rubrobacterales* (0–24%) and *Streptomycetales* (0–21%) and their respective taxa *Rubrobacter xylanophilus* (0–13%) and *Streptomyces scabiei* (0–6%) were also found in high abundance. *Proteobacteria* (22–47% RSA) were mainly represented by the orders *Enterobacteriales* (0.6–38%) and *Burkholderiales* (0.6–17%) and the taxa *Klebsiella pneumoniae* (0.1–32%) and *Salmonella enterica* (0–16%). *Firmicutes* (2–12%) were mainly represented by the order *Bacillales* (1–24%), and the taxa *Staphylococcus aureus* (0.3–34%) and *Cyanobacteria* (0–11%) mainly comprised *Cyanobacteriales* (0–14%) and *Nostocales* (0–10%). The remaining four phyla—*Bacteroidetes*, *Deinococcus Thermus*, *Planctomycetes*, and *Gemmatimonadetes*— exhibited minimal RSAs (0–2%). The most common taxa were mesophilic bacteria, with a minority being *thermophilic bacteria*. As expected, all the phyla appeared in the outgroup samples, for which we used soil from the vicinity of the Tel Megiddo, with RSAs that fit within the range above, albeit with high variation between the sites. Of the eight most common phyla, *Actinobacteria*, *Proteobacteria*, and *Firmicutes* inhabited all material surfaces (Figure 3B and Supplementary Table S4B). The remaining five taxa were present in all the materials except plastic, with *Bacteroidetes* also absent from cement.

### Microbial communities vary across areas and surface materials

To evaluate the microbial diversity in Tel Megiddo and how it varies between different areas, surface material, and public accessibility of the areas, we next compared the alpha diversity (within-sample diversity), beta diversity (similarity between communities), and composition within each variable for the top eight phylum levels that capture over 99% of the annotated phyla present in our samples (Figure 3).

The alpha diversity was reported using the number of observed species and the Shannon and Simpson indices (Supplementary Figure S2), reflecting each sample’s community richness and evenness (Supplementary Table S6). The number of species found in the samples ranged from 11 to 3,193, with a median of 780, had a Shannon index of 2.07–6.70 and a Gini-Simpson index of 0.77–0.996 (Supplementary Table S6 and Supplementary Figure S2), indicating high bacterial biodiversity. Area DD had the lowest number of species (*N* = 547), and area J had the highest number (*N* = 9,089) (Supplementary Table S6). The microbiomes exhibited significant differences across the studied areas (Kruskal-Wallis rank sum test: *p*-values are 0.093 for the observed species test, 0.037 for the Shannon index, and 0.020 for the Simpson index). Area S had the highest mean Shannon index and Simpson index, with 6.49 and 0.994, respectively, indicating the highest microbial diversity (Figure 3C; Supplementary Figure S3B). However, not all the pairwise groups had significant differences in the alpha diversity (observed species, Shannon index, and Simpson index; *Wilcoxon test*, *p*-value > 0.05; Figure 3C; Supplementary Figures S3A,B), which could have been an artifact owed to the small group sizes. The two-chambered city gate samples had the lowest microbial alpha diversity (Figure 3C; Supplementary Figures S3A,B) and showed the highest beta diversity distance from samples collected from other areas (Figure 3D and Supplementary Figure S3C).

Next, the beta diversity was calculated using the Bray-Curtis distance-based principal coordinate analysis (PCoA) (Figure 3D) and non-metric multidimensional scaling (NMDS) analysis (Supplementary Figure S3C). To address the concerns raised by Elhaik (2022) about the robustness of PCoA, we resampled 80% of each dataset 1,000 times and reported the mean and standard deviation of *R* and *p*. In the Bray-Curtis distance-based PCoA (Figure 3D) and NMDS (Supplementary Figure S3C) analyses (*stress* = 0.13), observed at 68% confidence intervals, the two-chambered city gate, northern Stables, and Area K showed more significant differences in taxa biodiversity than other areas despite the smaller sample size. The PERMANOVA analysis of beta diversity showed differences between the areas and materials, suggesting that the location of each area and its material composition influence microbial diversity.

Finally, samples were analyzed by whether or not they were collected from publicly accessible areas (Supplementary Table S1). The results of the microbial community’s alpha and beta diversity analyses, as well as taxonomical assignments, indicated no significant differences between these two groupings (Supplementary Figure S6). The difference remained insignificant even after controlling for the materials.

### Tel Meggido hosts diverse microbiomes that deviate from the microbiomes of the built environment

To study the extent to which the Tel Megiddo microbiomes resemble the microbiomes of the urban environment, we compared the RSA of Tel Megiddo and MetaSUB microbiomes with other cities in the Monumentome dataset.

#### Comparing the Tel Megiddo and MetaSUB microbiomes

**Figure 4:**
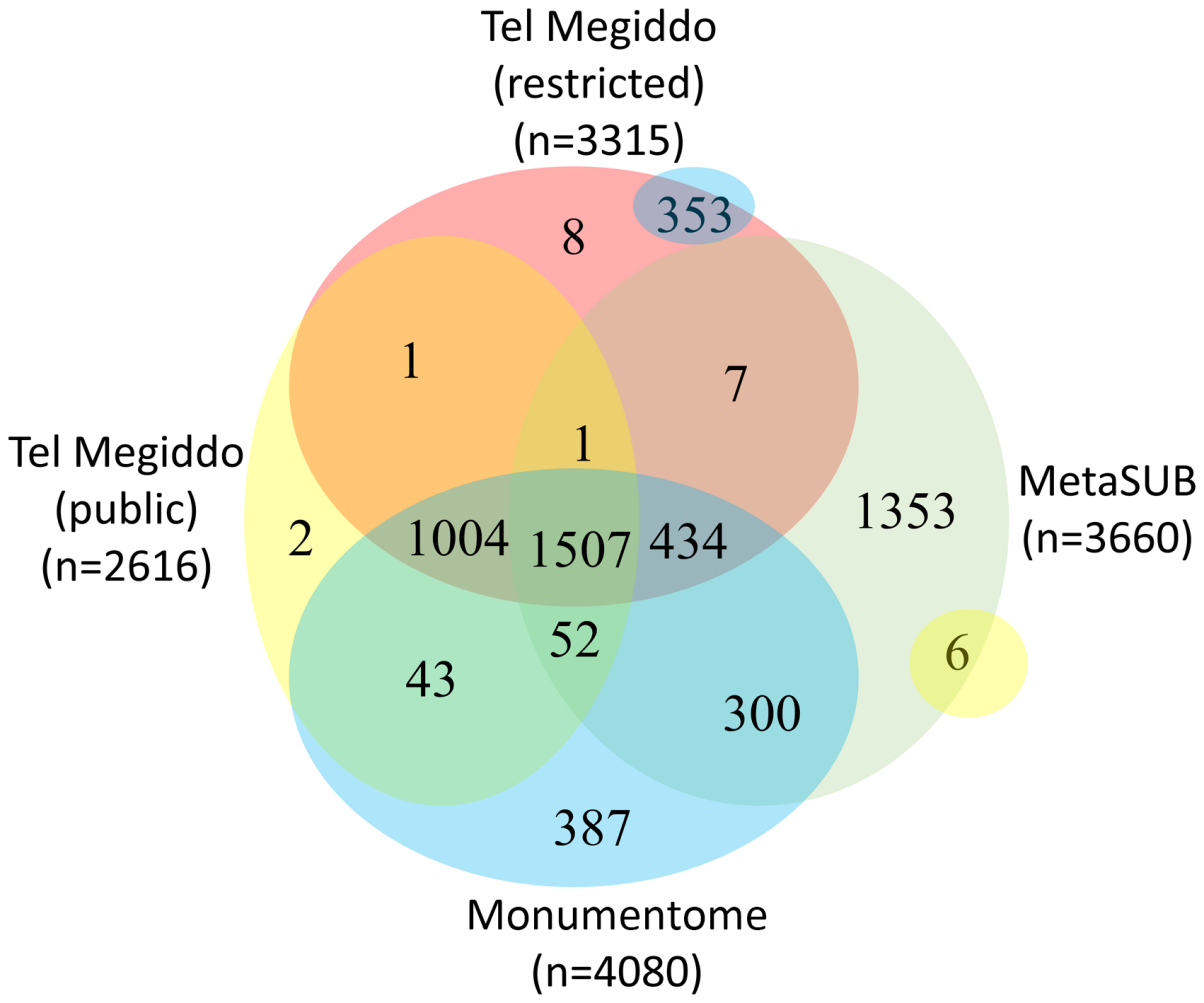
Venn diagram showing the taxa overlap among Tel Megiddos’ public and restricted sites, MetaSUB, and the Monumentome projects. The small yellow and blue bubbles represent extensions of intersections in other parts of the plot.

There were 1,411 taxa in Tel Megiddo that are absent from the MetaSUB dataset (Danko et al., 2021), 11 of which were also missing from all other studied monuments, with nine being exclusive to a single area within Tel Megiddo (Figure 4, Supplementary Table S7). Additionally, 2,007 taxa were common to Tel Megiddo and the MetaSUB dataset. Among those, 1,949 taxa (97%) with an RSA of 77% were sampled from Tel Megiddo’s publicly inaccessible sites, while 1,566 taxa (78%) with an RSA of 74% were sampled in the restricted site (Figure 4).

#### Comparing the Tel Megiddo and the Monumentome microbiomes

There were 3,393 taxa common to Tel Megiddo and other monuments. Of the 3,393 taxa common to Tel Megiddo and other monuments, 3,298 (97%) appeared in Tel Megiddo’s restricted areas with an RSA of ~100% and 2,606 (77%) in the publicly accessible areas with an RSA of ~100%. These findings indicate that the microbiomes in archeological sites partially overlap with the microbiomes of the built environment and that archeological and monument sites share similar microbiomes. We observed a few differences between publicly accessible and restricted areas at Tel Megiddo (Figure 4).

**Figure 5:**
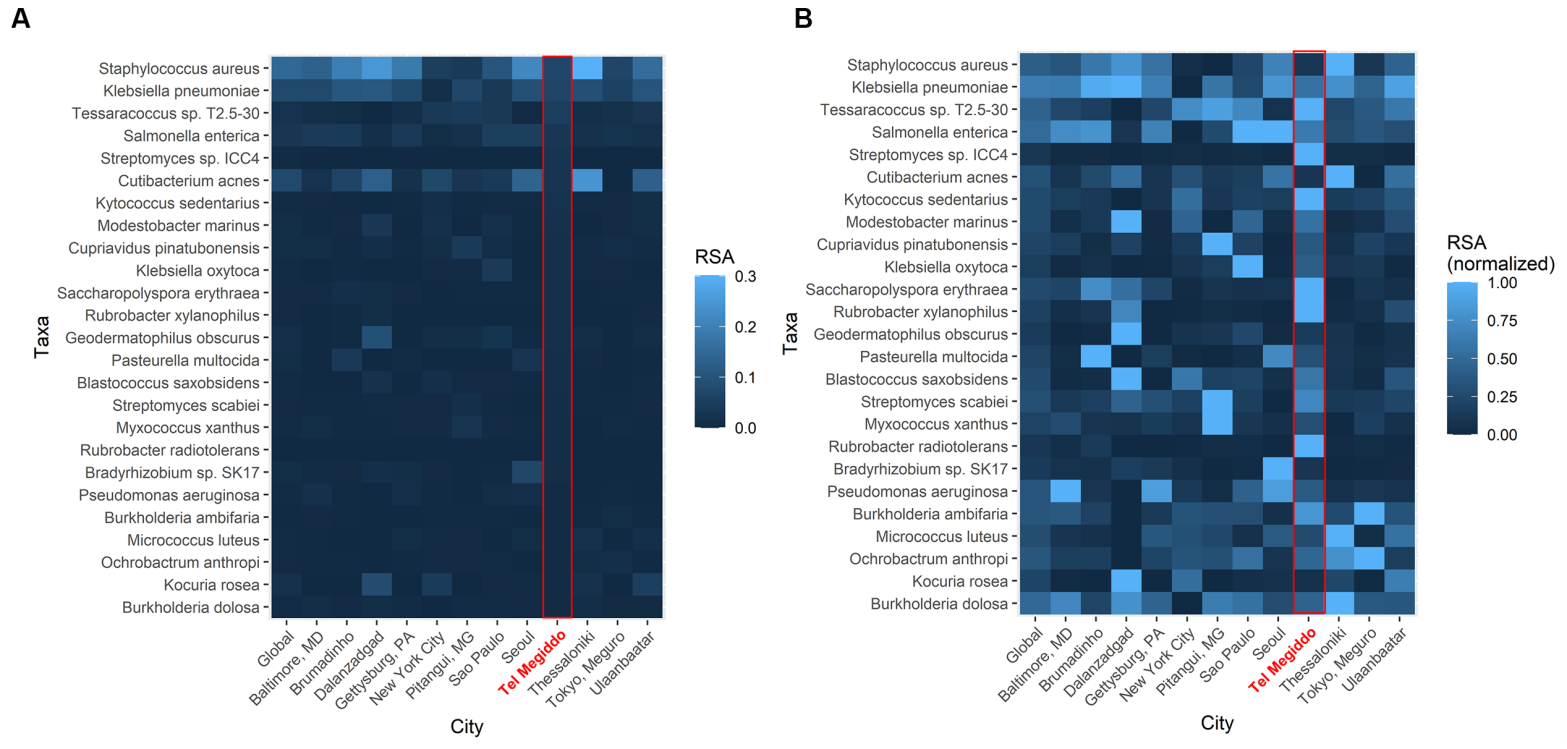
Geographic variations in the average abundance of the top 25 most common species-level taxa in Tel Megiddo observed across monuments in 12 cities. **(A)** The average RSA of these taxa in each city. **(B)** The average RSA of each taxon is normalized to account for variations between cities. Normalized abundance values are depicted on a scale from 0 (very dark blue) to 1 (bright blue), with the latter indicating higher taxon abundance in comparison to other cities.

To further investigate the similarity of these sites, we compared the RSAs of the 25 taxa with the highest RSAs in Tel Megiddo with those collected through the Monumentome Project, a dataset of nearly 4,100 microbiomes from 21 worldwide monuments in 12 cities. In Tel Megiddo, some taxa, despite already being among the most enriched microbes here, had a lower RSA (range: 0.005–0.069) (Figure 5A) than in the other monuments. After RSA normalization, these microbes showed different patterns of RSA in different cities, with taxa like *Rubrobacter radiotolerans* featuring much higher RSAs than other cities (Figure 5B). We also identified *R. tropicus* (*Rubrobacter* sp. *SCSIO 52909*). In other words, taxa are extensively shared between monuments and define the microbiome of these unique environments. Furthermore, each site has unique RSAs for these 25 abundant taxa, which supports their potential utility as geographical biomarkers. Examining the Tel Megiddo taxa with respect to taxa identified in other monuments, 25 species-level taxa existed only in Tel Megiddo, and of those, 20 unique taxa appeared in only one area (Supplementary Table S7).

### Tel Megiddo hosts acid-producing bacteria (APB)

Microorganisms can damage monument surfaces through various means, like biophysical (e.g., corrosion), biochemical (e.g., foul odor), and esthetic biodeterioration (e.g., stain), which may occur simultaneously or separately (Warscheid and Braams, 2000; Gil et al., 2015; Mihajlovski et al., 2017; Valentin, 2017). Members of the deleterious acid-producing bacteria of the *Acidobacteria* phylum were found in all areas of Tel Megiddo, albeit in small quantities (*Acidobacteria* $\tilde{RSA}$ =0.0015). It is well established that in association with microscopic fungi, *Cyanobacteria*, one of the most abundant phyla at the site, contributes to the formation of black scabs responsible for esthetic damage (Gaylarde et al., 2007; Salvadori and Municchia, 2016). Finally, members of the *Propionibacteriales* order, which includes taxa capable of synthesizing propionic acid—an odoriferous, colorless, oily liquid—were also abundant ( $\tilde{RSA}$ = 0.08) (Supplementary Table S4A).

### Tel Megiddo hosts pathogens

We found 242 animal and plant pathogen taxa in Tel Megiddo (Supplementary Table S5B). Considering pathogens by public accessibility, 228 animal and plant pathogenic taxa were found in samples collected from restricted sites with a total average RSA of 13.1%, while 164 pathogenic taxa were found in samples collected from publicly accessible sites with a total average RSA of 12.5% (Supplementary Table S5D). Interestingly, after controlling for material type, the average RSA of pathogenic taxa was higher in the samples collected from public sites than those collected from restricted sites (Supplementary Figures S5, S6E), but the difference was not significant (Mann–Whitney *U* test, *p*-value > 0.05).

The publicly accessible sites Area CC (the southern palace) and the two-chambered city gate have the highest pathogen RSA (Supplementary Figure S4A). These popular tourist sites may serve as a biodiversity hub. Area K and the Assyrian Palace, both restricted to visitors, had the least RSA of pathogens. Area J had the most animal pathogens, followed by Area S and the stables. Area J also had the most plant pathogens, followed by Areas S and K. The most common potential pathogens in Tel Megiddo, according to the sum of RSA across sites, were *Klebsiella pneumoniae* (41%), which can cause pneumonia and other infections; *Salmonella enterica* (29%), the most common pathogen causing food poisoning; and *Klebsiella oxytoca* (13%) (Supplementary Table S5B), an emerging multi-drug resistant pathogen. Both Areas CC and K harbored a similar number of burial sites (16 and 15, respectively), which cannot explain the different pathogen counts. But unlike Areas S and K, which are less accessible, Area CC has been actively explored by international archeologists. The accessibility of the site to visitors and archeological teams may explain the different pathogen counts, but larger sample sizes are required to confirm this.

Concerning the presence of pathogenic taxa across materials, interestingly, most pathogens were found in basalt, which had 220 pathogens out of a total of 242 found in all materials, followed by stone (170) and soil (160), with the least found in plastic (5). The total pathogen RSA was highest in cement (0.21), followed by wood (0.18), and the lowest in the soil and ceramics (0.07 and 0.08) (Supplementary Figure S5; Supplementary Table S5C).

## Discussion

### Tel Megiddo microbiome comprises lineages typical to arid regions

To study the microbial communities at Tel Megiddo, we carried out a metagenomic analysis of whole-genome shotgun metagenomic sequencing data and identified eight major phyla that dominate the microbiota at the site (Figure 3A). These phyla are similar to those of a survey of bacterial communities residing on limestone rocks in the Negev Desert (Israel) in the summer, which included *Cyanobacteria*, *Actinobacteria*, *Proteobacteria*, *Bacteroidetes*, *Chloroflexi*, *Deinococcus Thermus*, *Planctomycetes*, and *Gemmatimonadetes* (Nir et al., 2019). *Chloroflexi* were also found in Tel Megiddo but at very low RSA. Likewise, *Cyanobacteria*, which had an RSA of over 50% in the Negev Desert during the summer and 30% during the winter, had an RSA of ~5% in Tel Megiddo. *Cyanobacteria* were found mainly on the basalt (~20%), with relatively low RSA on ceramics and stones. We speculate that this is because the surfaces of the basalt rocks in Tel Megiddo are poorer in organic nutrients like nitrogen, phosphorus, and carbon, essential for plant and microbial growth [Galilee basalt consists mainly of plagioclase and pyroxene (Oppenhein, 1964)]. By contrast, limestones can be relatively rich in organic nutrients. Their calcium carbonate provides a source of calcium and buffers pH levels, providing a favorable environment for nutrient cycling (Adams et al., 1967). Moreover, the samples were taken from the limestones above the grounds, with some positioned vertically, whereas the limestone rocks in the Negev are on the ground, and their surface can attract plant debris and animal feces that enrich the nutrient content of the rock surfaces. Microbiome analysis of the Lincolnshire (England) limestones has also yielded a moderate RSA of *Cyanobacteria* (6–13%) (Skipper et al., 2022). Based on phyla-level, the RSA of the taxa found in that survey also resembles our results (ordered by RSA): *Actinobacteria*, *Proteobacteria*, *Cyanobacteria*, *Firmicutes*, *Bacteroidetes*, *Acidobacteria*, and *Deinococcus Thermus*. We are aware that phylum-based comparisons are coarse. However, the study by Skipper et al. (2022) did not include taxonomic levels below phylum to allow a more detailed comparison with our findings.

### Tel Megiddo hosts diverse microbial communities

Identifying taxa unique to Tel Megiddo with respect to the MetaSUB and Monumemtom datasets demonstrates that more taxa remain to be discovered in sites that exhibit outliers of the urban environment. Overall, the high alpha diversity indices and the uniqueness of the Tel Megiddo taxa suggest that the Tel Megiddo microbiome represents a complex community with high functional diversity. This diversity is not surprising, provided the various materials on the site and the continuous influx of microbiomes from international visitors. Thus far, visitors do not appear to negatively affect the ecosystem considering its high similarity to similar ecosystems, like the Negev desert, although we caution that more detailed comparisons at the level of community and function are not available due to the use of 16S rRNA and lack of whole genome data in the former study.

### Tel Megiddo’s microbiome is more similar to the monumentome microbiome than to the microbiome of the built environment

The site of Tel Megiddo is a unique combination of a built environment and a natural environment, thus potentially combining the microbiomes of these disparate ecosystems. A key question was whether the microbiome on the site resembles the microbiome collected from the built environment in the MetaSUB project (Ryon et al., 2022) or the microbiome of similar sites collected through the Monumentome Project. We found that the Tel Megiddo microbiome resembles the Monumentome microbiome more than the built environment. We speculate that this is because the Monumentome Project also sampled from parts of the built environment that do not come in contact with humans, as opposed to the MetaSUB project, where samples were obtained from transit stations that always come in frequent contact with humans. These results indicate that the built environment found in nature has its unique microbial signature with components of the urban and natural environment.

### Tel Megiddo’s microbiome comprises lineages that can damage buildings

Acid-producing bacteria (APB) pose a risk to archeological sites by decreasing the pH of the microbe environment. Biocorrosion or microbiologically influenced corrosion (MIC) was shown to contribute to corrosion, loss of material, and eventually severe and irreversible damage to stone monuments, destruction of artifacts, and dissolution of the stone matrix, especially in the case of calcareous rocks (Douglas and Beveridge, 1998; Warscheid and Braams, 2000; Zimmermann et al., 2006). Conservationists are increasingly aware of the importance of identifying APB in archeological sites and treating the infected areas to prevent structural damage (e.g., Jroundi et al., 2020).

The most abundant bacteria of the top four phyla found at Tel Megiddo were heterotrophic (*Actinobacteria*, *Proteobacteria*, and *Firmicutes*) with a minority of autotrophic (*Cyanobacteria*) (Figure 3A) – all phyla comprise lineages that contribute toward surface corrosion through various mechanisms, including biofilm formation, color alteration, patina formation, crust formation, bioweathering as a consequence of calcium uptake, precipitation of calcium salt and formation of secondary minerals, whitish gray powder, patinas, white salt efflorescence, hyphae penetration in the painted layers resulting into pitting, detachment, cracking and loss of the paint (reviewed in Dakal and Cameotra, 2012). These factors may threaten the long-term preservation of the site.

It is noteworthy that in Tel Megiddo, there is a high abundance of the *Rubrobacter* genus (Figure 5B), specifically *R. radiotolerans* and *R. tropicus*. The latter was recently isolated from deep-sea sediment of the South China Sea (Chen et al., 2020). A total of 1.28 M reads were mapped to *Rubrobacter* in all sites of the Monumentome Project. The four locations with the most *Rubrobacter* reads (58% of the total reads) were situated at Tel Megiddo. The fifth location was Brazil, with 5% of the read count. Not much is known about *R. tropicus*, but it is a free-living, moderate thermophile, phylogenetically closest to *R. radiotolerans*. The read counts of *R. radiotolerans* are highly correlated between our dataset and the Monumentome dataset (*T-test*: *n* = 438, *r* = 0.98, *p* = 0.04). Of *R. radiotolerans’s* 330 K reads in all sites of the Monumentome Project, Tel Megiddo occupied the top three places with 54% of the reads, followed by a site in Baltimore, Maryland, with 5% of the reads. *R. radiotolerans* releases bacterioruberin and monoanhydrobacterioruberin, two carotenoid pigments (Saito et al., 1994) associated with stains and discoloration of monuments (e.g., Schabereiter-Gurtner et al., 2001; Ortega-Morales et al., 2004; Mihajlovski et al., 2017) and wall paintings (Imperi et al., 2007). In all of the above cited studies, *R. radiotolerans* was the most abundant species suspected of causing the damage. Other *Rubrobacter* species (e.g., *R. bracarensis*) were also isolated from stained structures (Jurado et al., 2012). Another *Rubrobacter* of interest found in high proportion in Tel Megiddo is *R. xylanophilus*. This thermophilic organism can degrade cellulose and hemicellulose, the major components of plant material, which may also cause staining where plant material has accumulated. Interestingly, the genus *Rubrobacter* was reported to be transferred between sites in the Eastern Mediterranean via dust storms (Gat et al., 2017).

These results suggest that precautions should be taken to avoid staining damage to the monuments at Tel Megiddo, including monitoring the site and further in-depth studies to determine the impact of these microorganisms. Future studies could also include microbial cultivation and strain-level characterization to determine the biological functions of specific populations.

### Tel Megiddo microbiome comprises key human pathogens

Several potential pathogens were found in Tel Megiddo, including *Klebsiella pneumoniae*, an increasingly crucial bacterial pathogen capable of causing severe organ and life-threatening disease. *K. pneumoniae* is normally found in the human intestines. Exposure to the bacteria (e.g., through respiration) may cause pneumonia (typically bronchopneumonia or bronchitis) (Russo and Marr, 2019). Another pathogen was *Salmonella enterica*, a zoonotic pathogen of substantial concern to global human and animal health. Following ingestion (e.g., through food), *S. enterica* invades the intestinal epithelium in the ileum and colon. There, it may either cause neutrophilic gastroenteritis or disseminate to systemic sites and cause sepsis (Knodler and Elfenbein, 2019). Finally, *Klebsiella oxytoca*, is an emerging bacterial isolate causing hospital-acquired infection in adults and having multiple drug resistance to commonly used antibiotics.

Interestingly, we found *Avibacterium paragallinarum*, a pathogen of chickens and birds (Guo et al., 2022), in Tel Megiddo. We note with interest that chicken bone is not one of the species reported in Tel Megiddo digs. These findings are dated to the time between the Middle Bronze III and Late Bronze I (MB III–LB I) (Sapir-Hen et al., 2017). However, this is not surprising as chicken bones typically preserve poorly. Future ancient metagenomic analyses may corroborate the existence of chicken on the site.

In summary, Tel Megiddo holds World Heritage status and attracts a large number of visitors. This is reflected in the microbiomes of Tel Megiddo, which combine microbiomes from arid regions and monuments with human pathogens, as revealed here for the first time. Our findings suggest that despite the presence of pathogens, the site likely does not pose a threat to visitors as long as standard hygienic practices are applied. We recommend that health authorities monitor the site.

### Study limitations

Our study has several limitations. First, additional surveys conducted across seasons and over time are necessary to obtain a comprehensive view of the microbial communities at the site. Second, the sampling focused on the most common materials in the major areas, resulting in some materials being undersampled and the exclusion of other areas (e.g., V and W) from the survey. Third, follow-up studies should incorporate transcriptomic and proteomic analyses to validate the microbial activity of the reported and discussed lineages. In conclusion, further studies and plans are required to mitigate the potential microbial-induced degradation of the cultural relics at the site.

## Methods

### Sample collection and preparation

In July 2018, we carried out a comprehensive survey of the microbial communities at the archeological site of Tel Megiddo, Israel. We followed the ethical guidelines described in Shamarina et al. (2017) . We collected 40 samples from various above-ground surfaces located in nine areas and an outgroup area 50 meters outside of Tel Megiddo. The areas included the visitor entrance, Assyrian palace (Iron Age), Areas: DD (located immediately to the east of the Megiddo gates, the current surface dates to the Late Bronze Age), J (located in the Early Bronze temples area, samples were collected from the basalt offering table, limestone pillar base, and the round altar), K (samples were collected below the floors of different Middle Bronze II–III layers), S (samples were collected from different strata dating to the Middle Bronze I), CC – the southern sector of the mound (samples were collected from a late Iron I basin and from Iron II building blocks), northern stables (beneath the cobblestone pavement), and the two-chambered city gate of the Iron II (Figure 1 and Supplementary Table S1). All the samples were analyzed together with samples collected as part of the Monumentome Project (data were obtained with permission from the Monumentome Consortium,^3^). Tel Megiddo map was created using Matlab’s (V9.11) Mapping Toolbox (V5.2), R2021b.

Following the protocol of Danko et al. (2021), surface samples were collected and preserved using a flocked swab with a storage tube containing a buffer that is optimized for DNA preservation. We used individually wrapped Isohelix Buccal Mini Swab (MS Mini DNA/RNA Swab, Isohelix, Cat.: MS-02) paired with a barcoded storage tube (2D Matrix V-Bottom ScrewTop Tubes, Thermo Scientific, Cat.: 3741-WP1D-BR/1.0 mL), hereafter referred to as ‘matrix tubes,’ prefilled with 400 μL of a transport and storage medium suitable for both DNA and RNA (DNA/RNA Shield, Zymo Research, Cat.: R1100), hereafter referred to as ‘Zymo Shield.’ Once the surface was sampled, the swab was immediately placed into a matrix tube containing Zymo Shield and stored in a−80°C freezer until DNA extraction.

Sampling was done by swabbing various artifacts and surfaces, visible or newly uncovered, for which public access was available or restricted on the site. All the metadata were recorded for each sample during the collection process to ensure that as much contextual information as possible was captured. The project’s leader did the sampling over 3 days while wearing disposable latex gloves prior to sample collection. The swab was dipped in the preservative medium for approximately 2 s before the swab was firmly dragged across the surface, using both sides and different angles, for 3 min to ensure the highest yield.

### DNA extraction and sequencing

Sample controls, DNA extractions, library preparations, next-generation DNA sequencing, and quality control checks were done as in Danko et al. (2021). Samples stored at −80°C were allowed to thaw to room temperature before performing a DNA extraction suitable for the transport and preservation medium used with the Isohelix swabs. The swabs were processed using the ZymoBIOMICS 96 MagBead DNA Kit (Zymo Research, Cat.: D4308).

The entire 400 μL volume of Zymo Shield, along with the Isohelix swab head, were transferred into a new tube containing a 0.6 mL dry volume of 0.5 mm and 0.1 mm lysis matrix (BashingBead Lysis Tubes, Zymo Research, Cat.: S6012-50), as well as an additional volume of 600 μL of Zymo Shield. Mechanical lysis using bead beating was performed on 18 samples at a time, using a Vortex-Genie 2 adaptor at maximum power for 40 min. A 400 μL volume of the resulting lysate in each tube was transferred into sterile 1.5 mL tubes (Eppendorf, Cat.: 022363204). DNA isolation was carried out using the ZymoBIOMICS 96 MagBead DNA Kit (Zymo Research, Cat.: D4308) according to the manufacturer’s instructions. Purified samples were eluted into 50 μL ZymoBIOMICS DNase/RNase Free Water. All samples were subjected to quality control (QC) through the Qubit 4 Fluorometer (Invitrogen, Cat.: Q33238) using the 1X dsDNA High Sensitivity (HS) assay (Invitrogen, Cat.: Q33231).

Library preparation for Illumina NGS platforms was performed at Weill Cornell Medical College using the Illumina DNA Prep Kit (Illumina, Cat.:20015828), as was previously described in Afshinnekoo et al. (2015). Briefly, this involved fragmenting with an LE Series Covaris sonicator (Woburn, MA) with a targeted average size of 500 nt, a bead clean-up step to remove fragments under 200 nt, A-tailing, adaptor ligation, PCR amplification, bead-based library size selection, and a final clean-up step. A BioAnalyzer 2,100 (Agilent, Cat.: G2939BA) was used to ensure libraries fell within a range of 450–650 bp. The resulting libraries were also subjected to QC before proceeding with sequencing.

The libraries were sequenced on an Illumina NovaSeq 6,000 (Illumina Inc., San Diego, CA) using Illumina NovaSeq 6,000 Reagent Kits according to the manufacturer’s instructions.^4^ The sequencing was performed by Weill Cornell Medical College’s Genomics Resources Core Facility.

### Quality control and taxonomic profiling

We ran a standard metagenomic quality control pipeline (default settings, unless specified) prior to taxonomic profiling. We used bbtools (V38.92) (Bushnell, 2014), predominantly, starting with clumpify [parameters: optical = f, dupesubs = 2,dedupe = t], followed by bbduk [parameters: qout = 33 trd = t hdist = 1 k = 27 ktrim = “r” mink = 8 overwrite = true trimq = 10 qtrim = ‘rl’ threads = 10 minlength = 51 maxns = −1 minbasefrequency = 0.05 ecco = f] to remove adapter contamination, and tadpole [parameters: mode = correct, ecc = t, ecco = t] to remove sequencing error. Unmatching reads were removed using bbtool’s repair function. Bowtie2 (parameters: --very-sensitive-local) alignment to the HG38 human genome was used to remove potentially human-contaminating reads (Langmead and Salzberg, 2012). Following these steps, Kraken2 (Wood et al., 2019) was run to generate taxonomic profiles for each sample. Overall, we analyzed 4,257 taxa classified into 45 phyla. From hereon, all the work was done in R (version 4.0.3). Our R script is available via GitHub.^5^

### Assessing the antiquity of the microbiota

Metagenomic samples were taxonomically profiled with the KrakenUniq tool (Breitwieser et al., 2018) using a full, non-redundant NCBI NT database, the default database in BLAST (Altschul et al., 1990). The profiling includes splitting the whole-genome shotgun metagenomic reads into *k*-mers (30-mers in our case) and matching them against a pre-computed hash-table of *k*-mer abundances in all reference genomes available in the NCBI NT database. In this way, each metagenomic read is assigned to an NCBI taxon. All prokaryotic and eukaryotic organisms detected by KrakenUniq were filtered with respect to depth (>200 assigned reads) and breadth of coverage (>1,000 unique k-mers), which ensures a good balance between sensitivity and specificity of organism detection. To further validate the detected organisms, we performed a global (end-to-end) alignment of the DNA reads to the indexed NCBI NT reference genome with Bowtie2 (Langmead and Salzberg, 2012) and MALT (Herbig et al., 2016). The latter utilizes a Lowest Common Ancestor (LCA) algorithm that can deal with DNA reads mapping with the same affinity to multiple organisms. Next, we computed a number of quality metrics for authenticating the organisms detected in the metagenomic samples. The evenness of coverage was addressed by *samtools depth* (Li et al., 2009). Edit distance and deamination profile were calculated by *MaltExtract* tool from HOPS pipeline (Hübler et al., 2019). In addition, we computed read length distribution and post-mortem damage (PMD) score distribution via PMDtools (Skoglund et al., 2014). The ancient status of detected organisms was determined by inspecting the incremental pattern of transition mutation frequencies at the terminal ends of the DNA reads and the DNA fragmentation via reads length distribution. Based on the deamination pattern and the read length distribution quality metrics, we determined that very few to none of the sampled organisms were ancient.

### Estimating the relative sequence abundance of taxa (RSA)

We use the term *relative sequence abundance* (RSA) to describe the fraction of DNA in a sample from a given taxon. To determine the RSA of taxa (where applicable) in each profile, i.e., the fraction of DNA in a sample from a given taxon, we subsampled each sample to 100,000 classified reads and computed the proportion of whole-genome reads assigned to each taxon. We took the distribution of values from all samples. This was the minimum number of reads sufficient to maintain taxonomic richness. Sub-sampling was reported to effectively estimate RSA by Weiss et al. (2017). When we focused on the microbial community analysis for Tel Megiddo, we calculated RSA restricted to bacteria only. We discarded taxa with read numbers below 100, and calculated RSA by dividing the number of bacterial reads belonging to a respective taxon by the number of reads belonging to all bacterial taxa of that sample.

To compare the taxa RSA of Tel Megiddo with other cities, we also included archaeal, eukaryotic, and viral taxa with read numbers above 100. The average regional microbial RSAs (city) weighted by the sample size per region was calculated. Normalization of the RSA data of each taxon in each city was performed using Min-Max Normalization (Patro and Sahu, 2015).

### Sample annotation

The reads were taxonomically annotated by the Microbe Directory v2.0 (Sierra et al., 2019) and GTDB (Parks et al., 2021). Using the file bac120_metadata.tar.gz,^6^ we obtained the NCBI, GTDB, Greengenes, and SILVA taxonomy. The search was hierarchical. If the annotation was not found in NCBI, it was obtained from GTDB, otherwise from Greengenes, and finally from SILVA. Bacterial orders that appeared in more than one phylum were unified according to their recorded taxonomy in the Microbe Directory, NCBI, and GTDB (at that order). The pathogenicity of taxa was annotated by Microbiome Dictionary v2.0. The heterotypic synonym of microorganism were adjusted manually according to the their name in Microbe Directory, NCBI taxonomy and GTDB taxonomy (at that order).

We recorded the area, surface material, and access information per sample. Concerning alpha diversity indices, the numbers of observed species (richness) were calculated using the R function *estimateR*. Shannon-Wiener diversity and Gini-Simpson diversity indices were calculated using the function *diversity* in the R package “vegan” (version 2.5–7).

### Calculating the diversity of microbial communities

Differences in alpha diversity for the tested groups were assessed using the Kruskal-Wallis Rank Sum Test (applied by the R function *kruskal.test* in the “stats” package) and Wilcoxon signed-rank test (applied by the function *geom_signif* function in the “ggsignif” package). The beta diversity statistics PCoA and NMDS were calculated using the R function *BetaDiv* in the “amplicon” package (Liu et al., 2021). The distance matrix between samples was calculated based on Bray-Curtis dissimilarities. Differences in beta diversity for the tested groups were assessed by permutational multivariate analysis of variance in PERMANOVA using the R function *adonis* in the “vegan” package. We calculated prevalence as the number of times each microorganism appeared at the sites, i.e., the percentage of sites where a given taxon was present with RSA higher than zero (Supplementary Table S6).

## Data availability statement

The data presented in the study are deposited in the GeoSeeq.com repository, accession name: Monumentome Project available at: https://portal.geoseeq.com/sample-groups/0c919d45-410e-4edc-8d0b-1529f6516726.

## Author contributions

YZ: Investigation, Methodology, Software, Writing – original draft, Writing – review & editing. SER: Methodology, Writing – review & editing. NO: Methodology, Writing – review & editing, Formal analysis, Investigation. BT: Formal analysis, Methodology, Writing – review & editing. KR: Writing – review & editing, Data curation. DD: Data curation, Writing – review & editing. CM: Data curation, Writing – review & editing, Supervision, Conceptualization, Funding acquisition, Resources. EE: Supervision, Writing – review & editing, Conceptualization, Funding acquisition, Investigation, Methodology, Project administration, Resources, Software, Visualization, Writing – original draft.

## Funding

The author(s) declare financial support was received for the research, authorship, and/or publication of this article. EE was partially supported by the Crafoord Foundation, the Swedish Research Council (2020-03485), Erik Philip-Sörensen Foundation (G2020-011). EE was a beneficiary of the Horizon Europe Project (101120165). The computations were enabled by resources provided by the Swedish National Infrastructure for Computing (SNIC) at Lund, partially funded by the Swedish Research Council through grant agreement no. 2018-05973. SER was supported by a grant from the Simons Foundation (824763). NO was financially supported by Knut and Alice Wallenberg Foundation as part of the National Bioinformatics Infrastructure Sweden at SciLifeLab.

## Conflict of interest

The authors declare that the research was conducted in the absence of any commercial or financial relationships that could be construed as a potential conflict of interest.

## Publisher’s note

All claims expressed in this article are solely those of the authors and do not necessarily represent those of their affiliated organizations, or those of the publisher, the editors and the reviewers. Any product that may be evaluated in this article, or claim that may be made by its manufacturer, is not guaranteed or endorsed by the publisher.

## Footnotes

^1^ https://ims.gov.il/en/ClimateAtlas

^2^ https://www.meteoprog.com/weather/Telmegiddo/month/may/

^3^ https://portal.geoseeq.com/sample-groups/0c919d45-410e-4edc-8d0b-1529f6516726

^4^ https://www.illumina.com

^5^ https://github.com/YaliZhang98/Tel-Megiddo

^6^ https://data.gtdb.ecogenomic.org/releases/release202/202.0/
