# Supplementary material for: The microbial biodiversity at the archeological site of Tel Megiddo (Israel)
Source: Front Microbiol. 2023 Sep 22;14:1253371. doi: 10.3389/fmicb.2023.1253371 (PMC10559971; doi:10.3389/fmicb.2023.1253371)
Supplement: Supplementary file 1 [file Data_Sheet_1.docx]

Supplementary Materials

## Figure S1

**The taxonomic composition of Tel Megiddo.** Top: taxa count in each kingdom group. The numbers displayed in the figure represent the counts for each group. Of the 4,259 taxa in Tel Megiddo, 3,418 (80%) were bacteria. Bottom: Counts and percentages of bacterial taxa classified into phyla.


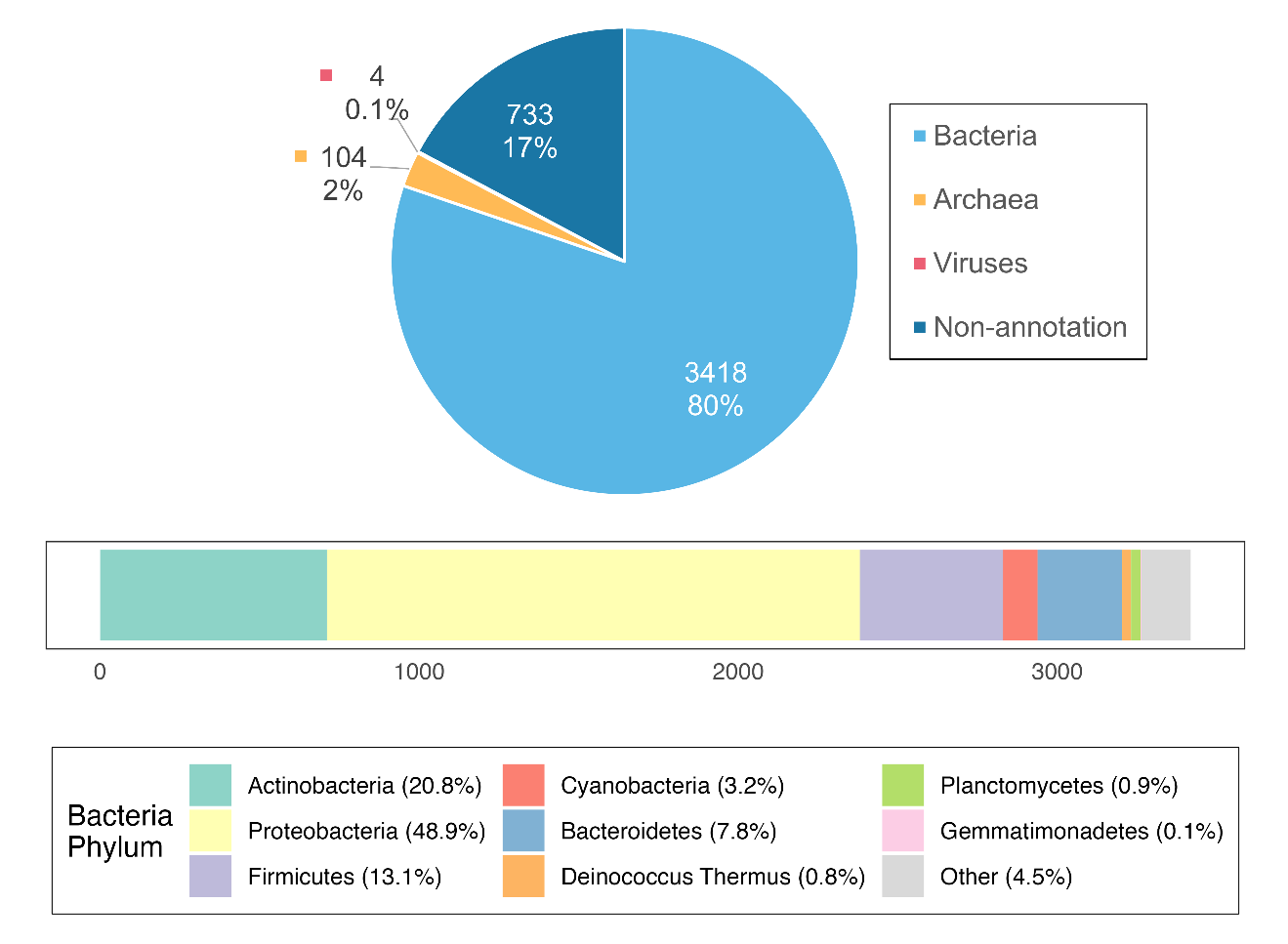


## Figure S2

**Alpha diversity index for the Tel Megiddo samples (*n*=40).** Bar plots show the observed species (A), Shannon index (B), and Simpson index (C) (Table S2).


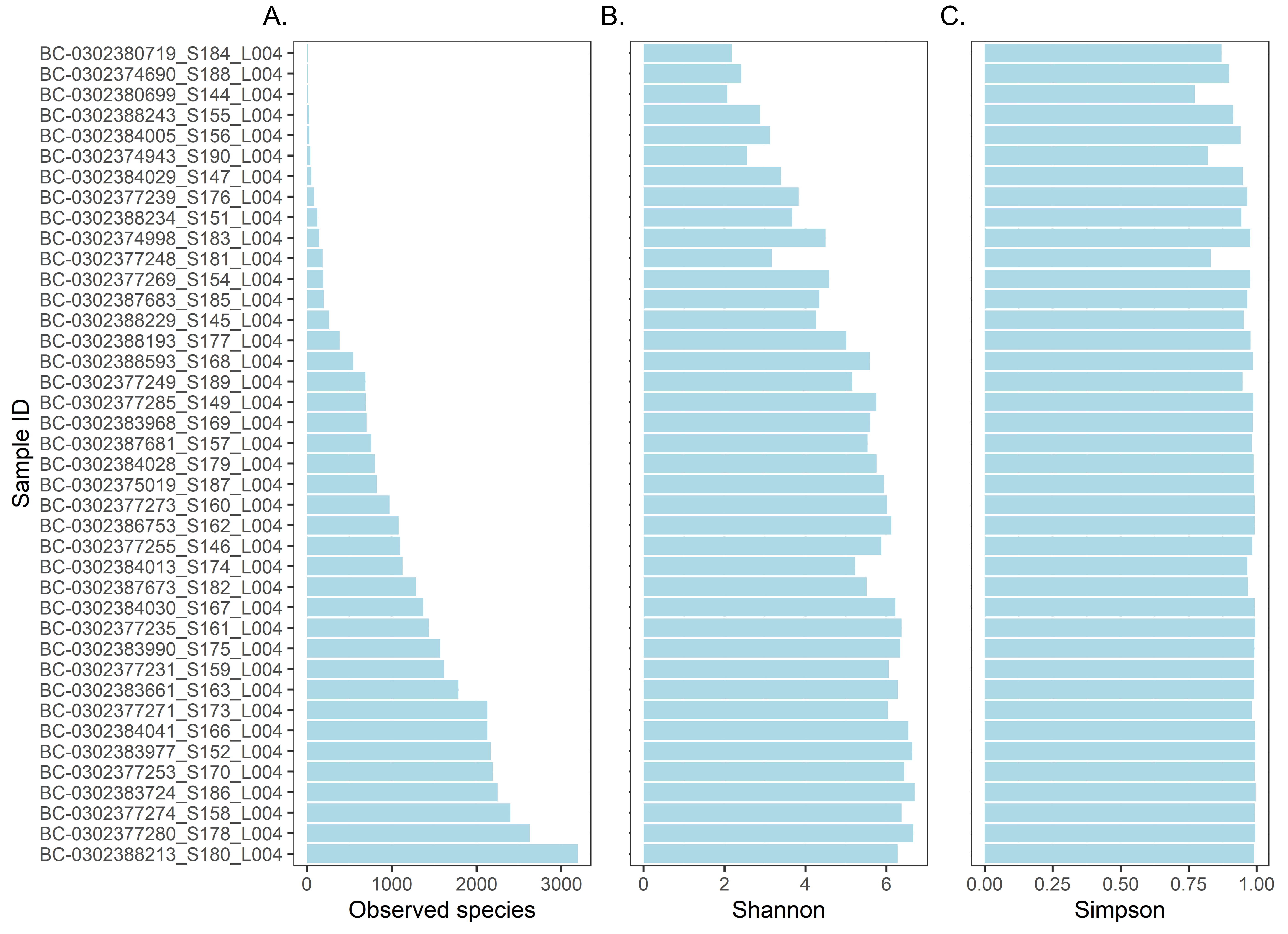


## Figure S3

**Microbial diversity of samples collected from different areas and materials at the phylum level.** The microbial community from different sites. The observed species and Gini-Simpson indices of the microbial community from different areas (A,B). Grouping of microbial communities according to their taxonomic compositional similarity (Bray-Curtis distances) using nonlinear multidimensional scaling in different areas. Statistical significance was assessed using the Wilcoxon signed-rank test. *P*-value are indicated in the plots as 0-0.001***, 0.001-0.01**, 0.01-0.05*, or NS (non-significant difference). The principal component analyses were carried out based on Bray-Curtis distances of the microbial communities in different areas (C) The *PERMANOVA test* yielded: *r*=0.38±0.03 and *p*=0.04±0.04. Clusters were drawn using the convex hull function.


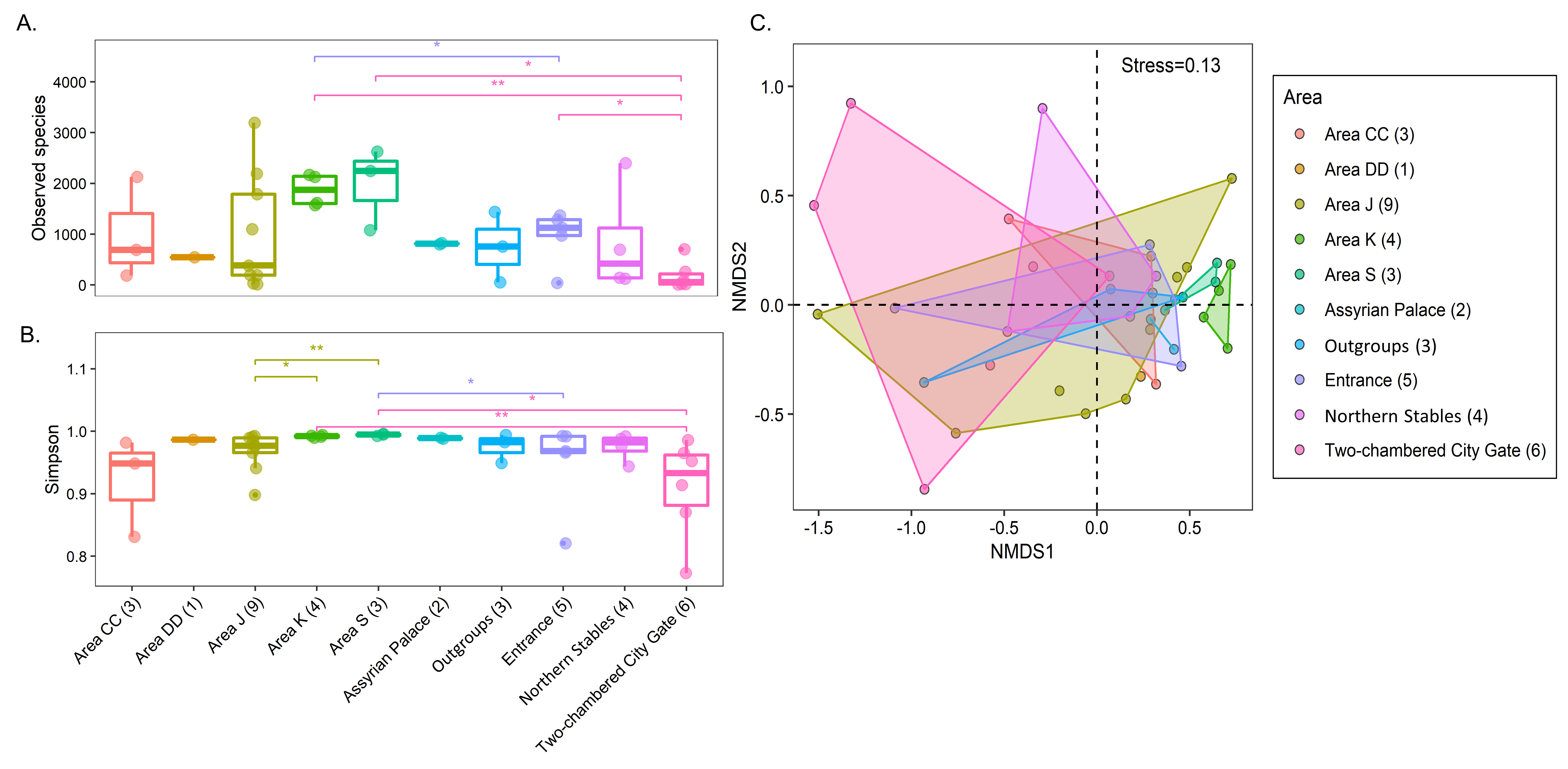


## Figure S4

**RSA of pathogens in different areas and materials at Tel Megiddo.** The RSA of animal and plant pathogens per site (A) and per surface material (B). Pathogens are color-coded by animal (red), plant (blue), and dual-kingdom (green) hosts.


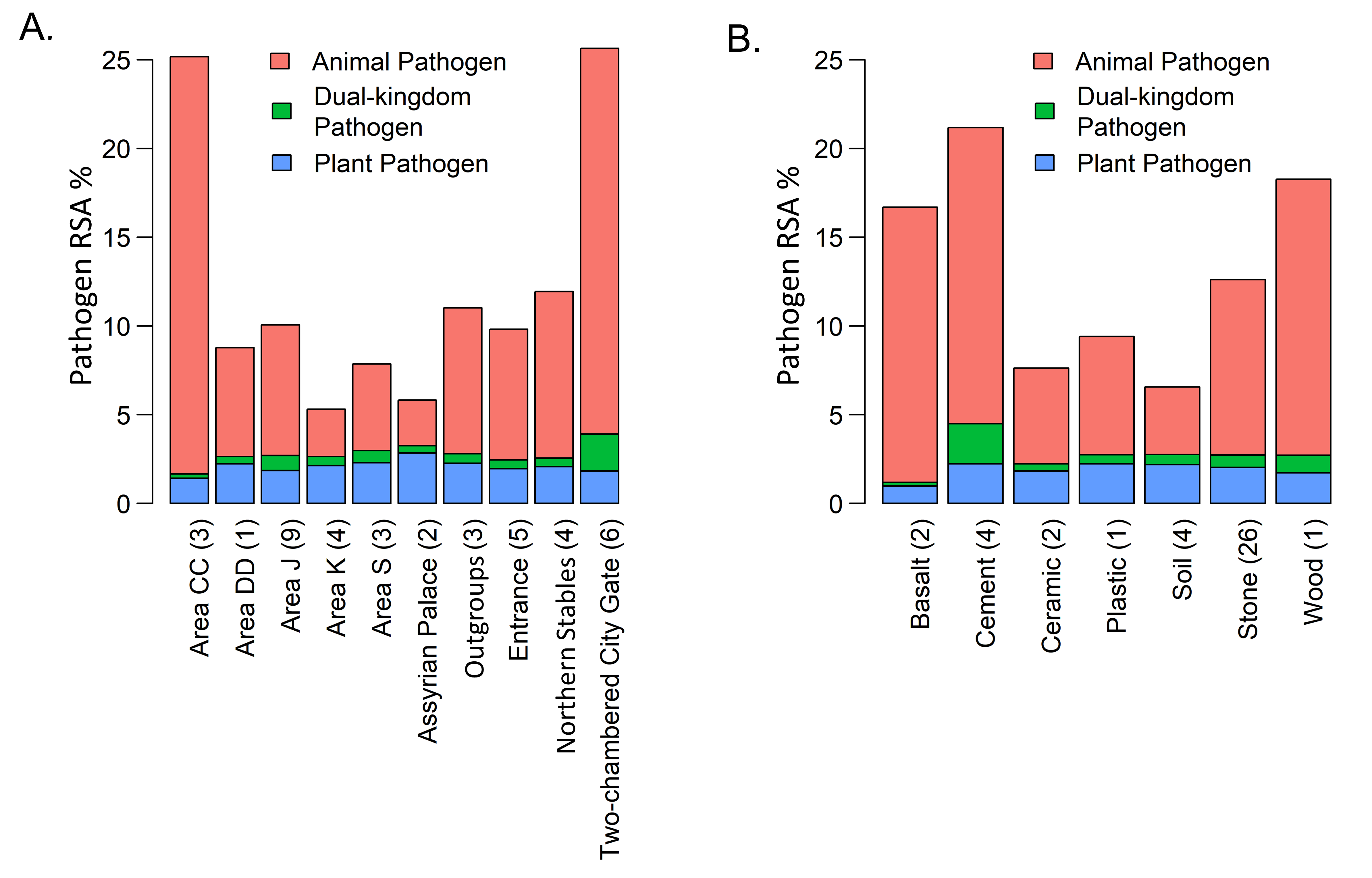


## Figure S5

**Microbiome composition and diversity in publicly accessible and restricted areas.** (A) The taxonomic composition of taxa at the phylum level. *Other* refers to all other phyla with lower RSA than the displayed phyla and *No annotation* refers to the taxa whose phylum could not be annotated. (B) The number of observed species in the microbial communities. (C) The Shannon indices of the microbial communities. (D) The Gini-Simpson indices of the microbial communities (E) The relative sequence abundance of pathogens. Principal component analysis (F) and non-metric multidimensional analysis (G) of community similarity based on Bray-Curtis distance matrices (PCoA *PERMANOVA test* yielded*: r*=0.03±0.007, *p*=0.38±0.15; NMDS *PERMANOVA test* yielded*: r*=0.03±0.007, *p*=0.37±0.15). Statistical significance was assessed using the Wilcoxon signed-rank test with the *p*-value marked in the plots as 0-0.001***, 0.001-0.01**, 0.01-0.05*, or NS (non-significant difference).


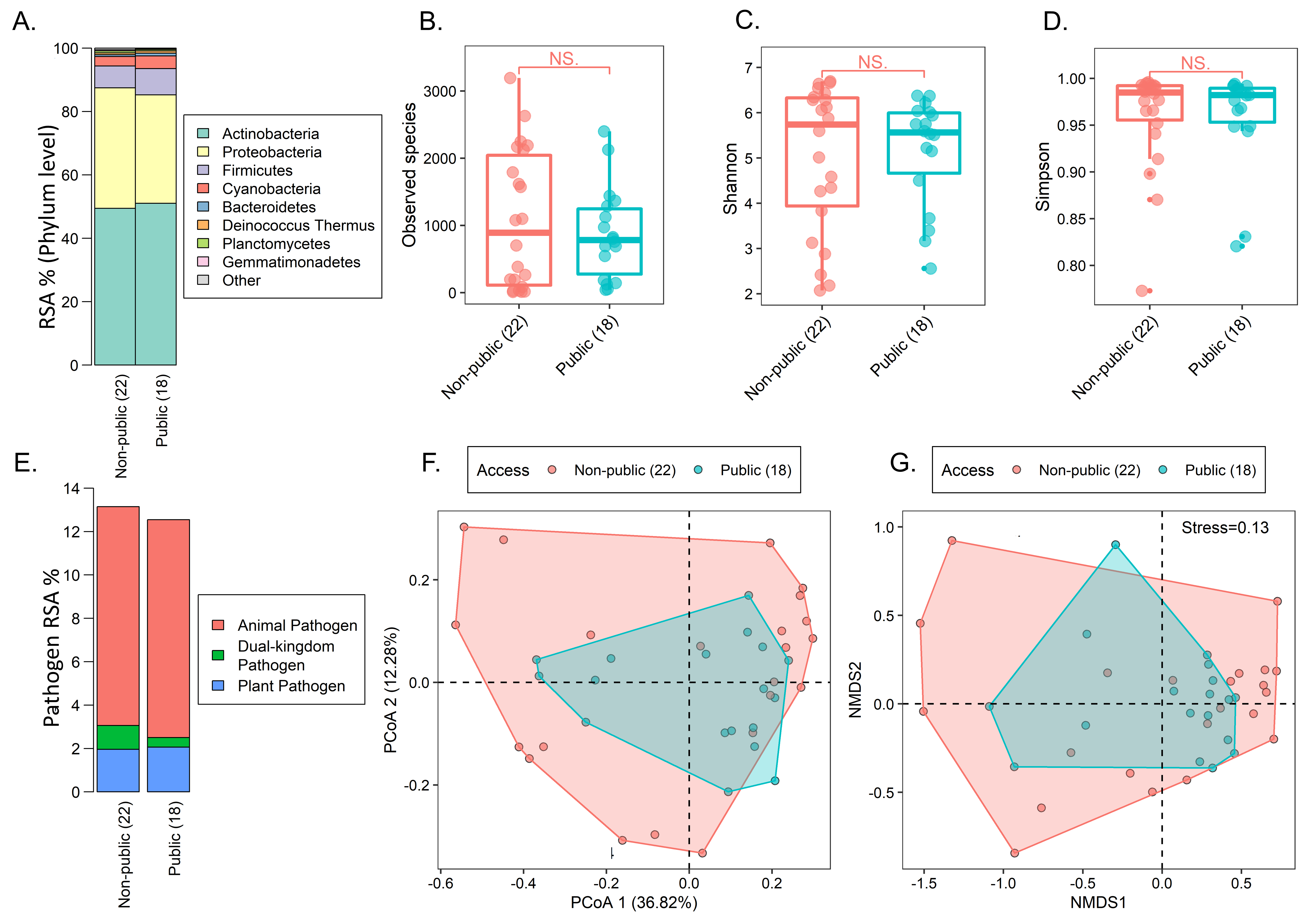


## Figure S6

**Microbiome composition and diversity in publicly accessible and restricted areas with controlled samples’ surface material.** Only samples from stone surface material are shown. (A) The taxonomic composition of phylum level of the taxa. *Other* refers to all other phyla with lower RSA than the displayed phylum, and *No annotation* refers to the taxa whose phylum could not be annotated. (B) The number of observed species in the microbial communities. (C) The Shannon index of the microbial communities. (D) The Gini-Simpson index of the microbial communities (E) The relative sequence abundance of pathogens. Principal component analysis (F) and non-metric multidimensional analysis (G) of community similarity based on Bray-Curtis distance matrices (PCoA *PERMANOVA test: r*=0.04±0.01, *p*=0.60±0.19; NMDS *PERMANOVA test: r*=0.04±0.01, *p*=0.60±0.19). Significance was assessed using the Wilcoxon signed-rank test with the *p*-value marked in the plots as 0-0.001***, 0.001-0.01**, 0.01-0.05*, or NS (non-significant difference).


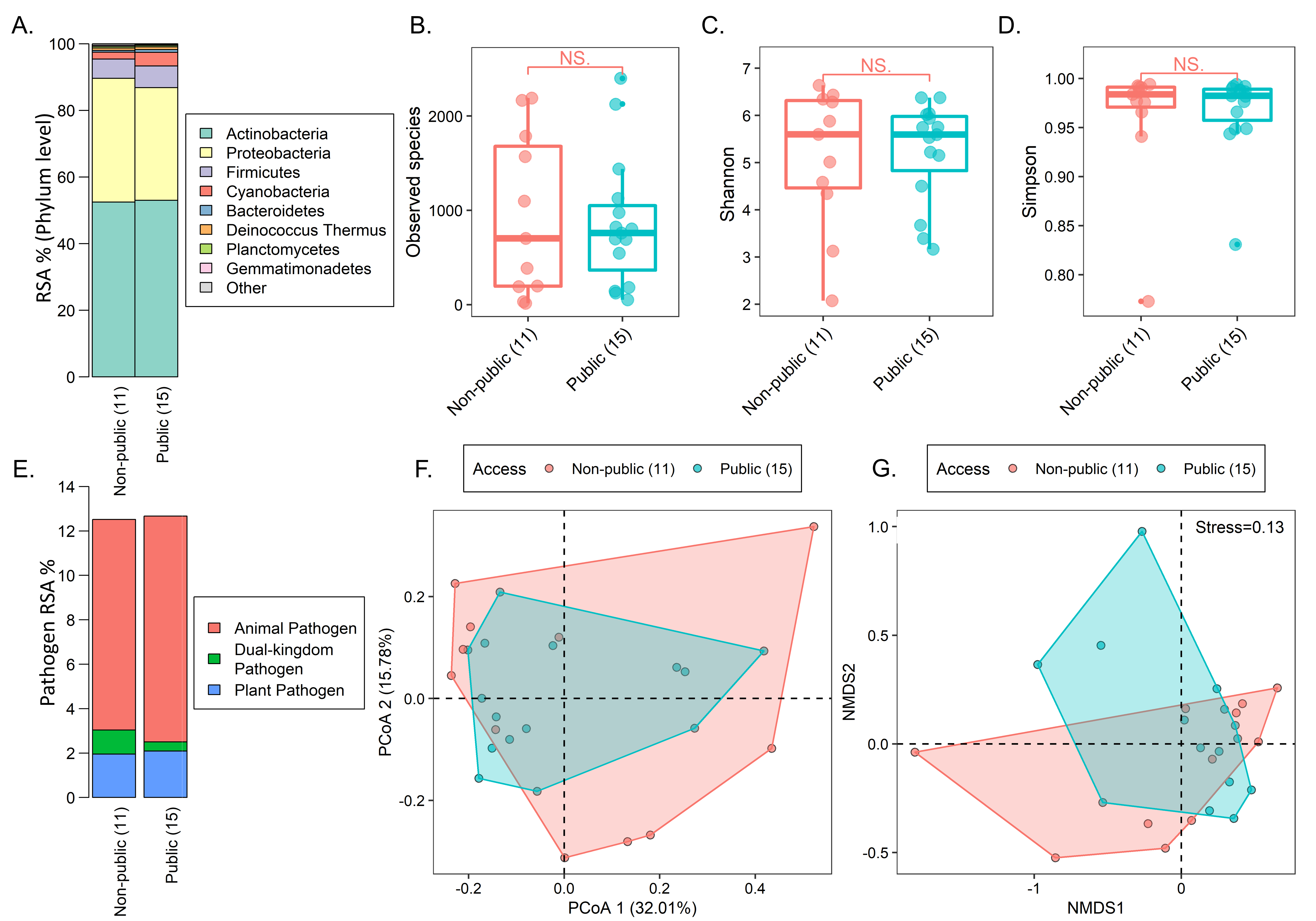


## Table S1

**MetaData of Tel Megiddo samples.** The column “Access” shows whether or not the site where the sample was taken is publicly accessible.

## Table S2

Relative Sequence Abundance **(RSA) by taxa.** RSA value of each bacterium in the samples collected in Tel Megiddo. The sum of the abundance values of all bacteria in each sample is one.

## Table S3

**Taxonomic annotation.** The taxonomic annotation of taxa collected at Tel Megiddo includes six taxonomic levels: Kingdom, Phylum, Class, Order, Family, Genus, and Species. Blanks indicate that the taxonomic annotation cannot be found. The column “Annotation_source” indicates the source of annotation (see Methods, *Sample annotation*). “Animal_pathogen” and “Plant_pathogen” indicate whether the taxa is an animal or plant pathogen (1=yes, 0=no, NA the pathogen annotation is missing from the Microbe Directory v2.0).

## Table S4

Relative Sequence Abundance (**RSA) by Phylum.** RSA value of each phylum in the samples collected in Tel Megiddo grouped by sites (a) and materials (b).

## Table S5

**Pathogens.** (a) Pathogen presence and annotation. The first three rows contain the number of pathogen species in each sample. The column “pathogen_type” is annotated with the type of pathogens. The presence of pathogens in the samples is marked in 1, with 0 marking their absence. RSA and summary for pathogens in each site (b), material (c), and access (d). The last six columns indicate the number of animal, plant, and dual-kingdom pathogens in each group along with the total RSA value. The last row, “pathogen type,” is annotated with the type of pathogens.

## Table S6

**Microbial alpha biodiversity.** “Read_number” indicates the read number for each sample. “Observed_species” indicates the number of bacteria in each sample. Communal biodiversity was estimated using the Shannon and Simpson indexes. “Goods_coverage” is the microbial coverage.

## Table S7

**Unique taxa.** Bacteria appear only in Tel Megiddo samples compared to other monuments. “Not shared in MetaSUB” indicates whether the bacterium is present in the MetaSUB database, and “Uniq taxa in Tel Meggido site” indicates whether (1) or not (0) the bacterium is present in only one site in Tel Megiddo.
